# Land snail fauna in Gunung Kuang Limestone Hill, Perak, Malaysia and its conservation implications (Mollusca, Gastropoda)

**DOI:** 10.3897/zookeys.769.25571

**Published:** 2018-06-26

**Authors:** Chee-Chean Phung, Yuen-Zhao Yong, Mohamad Afandi Mat Said, Thor-Seng Liew

**Affiliations:** 1 Institute for Tropical Biology and Conservation, Universiti Malaysia Sabah, Jalan UMS, 88400 Kota Kinabalu, Sabah, Malaysia; 2 Lafarge Cement Sdn Bhd, Batu 13 1/2 Miles, Jalan Kuala Kangsar, 31200 Chemor, Perak, Malaysia; 3 Rimba, 22-3A, Casa Kiara 2, Jalan Kiara 5, 50480 Kuala Lumpur, Malaysia

**Keywords:** Gunung Kanthan, karst conservation, Kinta Valley, Peninsular Malaysia

## Abstract

This paper presents the first land snail species checklist for Gunung Kuang (Kuang Hill), a limestone hill located next to Gunung Kanthan that is recognised as one of the most important limestone hills for its diverse land snail fauna in Kinta Valley. Samplings were carried out at five plots in Gunung Kuang. This survey documented 47 land snail species, in which six species were identified as unique to Gunung Kuang. Approximately half of the land snails from Gunung Kanthan were found in Gunung Kuang. In addition, one of six unique species from Gunung Kanthan was also found in Gunung Kuang. These rich land snail species in Gunung Kuang are similar to other hills in Kinta Valley, but it is relatively lesser than the adjacent Gunung Kanthan. In view of Gunung Kuang’s unique land snail species, and its location closest to disturbed Gunung Kanthan, Gunung Kuang should be considered in the conservation management plan for Gunung Kanthan.

## Introduction

Limestone hills are known for their rich biodiversity and endemism (Clements et al. 2006; Schilthuizen 2004). In particular, land snails are the most distinctive organism inhabiting the limestone hills (Clements et al. 2008; Foon et al. 2017; Liew et al. 2014; Schilthuizen et al. 2003). The state of Perak is one of the areas with a large number of limestone hills and a long history of land snail studies in Malaysia. This area has the highest recorded number of land snail species in Malaysia at present; ironically, it also has the largest number of operating quarries (Foon et al. 2017, Liew et al. 2016). Thus, a conservation plan is needed to mitigate the impact of the ongoing quarry on the land snail biodiversity in the limestone hills in Perak.

Among the limestone hills in Perak, Foon et al. (2017) found that Gunung Kanthan, a limestone hill gazetted for quarrying, has the highest number of land snail species and half a dozen of unique species. While a large part of Gunung Kanthan is being quarried away, the entire Gunung Kuang that is located 2 km away from Gunung Kanthan remains intact. Hence, documenting the land snails in Gunung Kuang is necessary to determine whether their species, especially the unique ones, can also be found in Gunung Kanthan. This information is important for planning and managing conservation efforts, such as making Gunung Kuang a possible alternative site for the conservation and rehabilitation of land snail species in Gunung Kanthan, if both hills share significant proportion of species diversity and composition.

In view of the above reasons, we surveyed the land snails in Gunung Kuang by using the same sampling technique applied by Foon et al. (2017). We reported a checklist of land snails that were found in Gunung Kuang and compared it with Gunung Kanthan and other limestone hills in Kinta Valley, Perak. Based on the results of the comparison, we discussed the implication on future conservation strategy for land snail fauna in the region.

## Materials and methods

### Study site

Gunung Kuang (N4.7467°, E101.1326°) is located within Kinta Valley and the south of Gunung Kanthan. Gunung Kuang is an intermediate-sized limestone outcrop measuring 0.3155 km^2^ (Liew et al. 2016) and is given a standardized national code of Prk 46 G. Kuang by Liew et al. (2016).

### Land snail sampling, processing, and identification

Five sampling plots measuring 2 × 4 m were selected to conduct sampling in Gunung Kuang (Clements et al. 2008; Liew et al. 2008; Foon et al. 2017). The locations of the sampling plots were: Plot 1: 4.742371°N, 101.132588°E; Plot 2: 4.742500°N, 101.133611°E; Plot 3: 4.742275°N, 101.133677°E; Plot 4: 4.742222°N, 101.133889°E; Plot 5: 4.74790944°N, 101.135°E. The sampling was conducted on 7^th^ October 2017 and 14^th^ February 2018. In each plot, living macro-snails and empty shells were searched and collected. In addition, five litres of top soil and leaf litters were collected, dried in an oven, and subjected through a series of sieves to extract micro-snails under a dissecting microscope in the laboratory.

Morphology-based species-level identifications were carried out by referring to the checklist of limestone land snails in Perak that was prepared by Foon et al. (2017) and comparison with specimens deposited in the BORNEENSIS Mollusca collection at the Institute for Tropical Biology and Conservation, located at Universiti Malaysia Sabah. Morphospecies that could not be assigned to an existing taxonomic name was given a provisional species name (e.g., *Microcystina* ‘Kuang 1’). For each of the newly recorded morphospecies recorded in this survey, photographs were taken using ZEISS EVO MA 10 scanning electron microscope. Photographs for previously recorded species are available in Foon et al. (2017). Species was listed as ‘unique’ if the species can only be found in one of the total limestone hills surveyed in Foon et al. (2017) and in this study. Every sample was given a collection number and catalogued into the BORNEENSIS database. In addition, all of the sampling materials were deposited into the BORNEENSIS collection at the Institute for Tropical Biology and Conservation.

## Results

This survey recorded a total of 47 land snail species belonging to 29 genera and 20 families from Gunung Kuang (Table 1). Among these species, six were unique to Gunung Kuang.

**Table 1.** Land snail species checklist found from Gunung Kuang and remarks.

Species	Remarks	Plot 1	Plot 2	Plot 3	Plot 4	Plot 5
**Achatinidae**						
*Achatina fulica* (Bowdich, 1822)		/				
**Ariophantidae**						
*Macrochlamys ‘Bercham 1*’	Foon et al. (2017)			/	/	/
*Macrochlamys ‘Kuang 1*’ *#	Medium-sized shell. Whorls and size similar to *Macrochlamys* ‘Batu Kebelah 1’ (Foon et al. 2017) but flatter spire without undulations of radial growth lines along the suture (Figure 5).		/		/	
*Microcystina clarkae* Maassen, 2000				/	/	/
*Microcystina ‘Kuang 1*’ *#	Small yellowish shell. Whorl, shape and size similar to *Microcystina* ‘kanthan 1’ (Foon et al. 2017) but shell surface glossy and apical whorls with spiral grooves (Figure 1).	/				
**Ariophantidae**						
*Microcystina ‘Kuang 2*’ *#	Small shell. Whorl, shape and size similar to *Microcystina* ‘tempurung 3’ (Foon et al. 2017) but shell surface with dense spiral grooves (Figure 2).	/				
*Microcystina ‘Kuang 3*’ *#	Small brown shell. Whorl, shape and size similar to *Microcystina* ‘tempurung 2’ (Foon et al. 2017) but with strong undulations of radial growth lines along the suture (Figure 3).	/				
*Microcystina sinica* Von Moellendorff, 1885		/				/
*Microcystina townsendiana* Godwin Austen & Nevill, 1879				/		
**Assimineidae**						
*Acmella ‘Kanthan 1*’	Foon et al. (2017)	/				
**Bradybaenidae**						
*Bradybaena similaris* (Férussac, 1821) *					/	
**Camaenidae**						
*Chloritis breviseta* (Pfeiffer, 1862)		/				
**Charopidae**						
*Charopa lafargei* Vermeulen & Marzuki, 2014				/		
*Charopa ‘Kanthan 1*’	Foon et al. (2017)	/				
**Clausiliidae**						
*Phaedusa filicostata kapayanensis* (deMorgan, 1885)*					/	
**Cyclophoridae**						
*Alycaeus kapayanesis* (De Morgan, 1885)		/	/			
*Chamalycaeus diplochilus* (von Moellendorff, 1886)		/		/		/
*Cyclophorus malayanus* (Benson, 1852)		/				
*Cyclophorus semisulcatus* (Sowerby, 1843)		/				
*Platyraphe lowi* (De Morgan, 1885)		/	/			/
*Rhiostoma jousseaumei* de Morgan, 1885		/	/	/	/	
**Diapheridae**						
*Sinoennea subcylindrica* (von Moellendorff, 1891)		/				/
**Diplommatinidae**						
*Diplommatina cf diminuta*	Foon et al. (2017)		/	/		
*Diplommatina crosseana* Godwin-Austen & Nevill, 1879		/	/			
*Diplommatina nevilli* Crosse, 1879		/	/	/		/
*Diplommatina superba superba* Godwin Austen & Nevill, 1879			/	/		
**Diplommatinidae**						
*Diplommatina ventriculus* (V.Moellendorff, 1891)				/		
*Opisthostoma paulucciae* (Crosse&Nevill, 1879)						/
**Dyakiidae**						
*Pseudoplecta bijuga* (Stoliczka, 1873)		/				
*Quantula striata* (Gray, 1834)			/			
**Endodontidae**						
*Philalanka pusilla* Maassen, 2000		/				
**Euconulidae**						
*Kaliella ‘Kuang 1*’*#	Small shell. Whorl, shape and size similar to *Kaliella barrakporensis* but shell surface without regular radial growth line.			/		
*Kaliella barrakporensis* (Pfeiffer, 1852)		/				/
*Kaliella doliolum* (Pfeiffer, 1846) *#				/	/	
*Kaliella microconus* (Mousson, 1865) *		/	/		/	/
*Kaliella scandens* (Cox, 1872)		/			/	
**Ferussaciidae**						
*Cecilioides caledonica* (Crosse, 1867) *		/		/	/	
**Hydrocenidae**						
*Georissa monterosatiana* Godwin Austen & Nevill, 1879		/	/	/	/	
*Georissa semisculpta* Godwin Austen & Nevill, 1879		/	/			
**Pupinidae**						
*Pupina tchehelensis* de Morgan, 1885			/	/	/	/
**Streptaxidae**						
*Discartemon plussensis* (de Morgan, 1885)			/		/	
*Gulella bicolor* (Hutton, 1834)			/	/		
**Subulinidae**						
*Allopeas clavulinum* (Potiez & Michaud, 1838)			/			/
*Allopeas gracile* (Hutton, 1834)			/			
*Prosopeas tchehelense* (De Morgan, 1885)		/				
**Vertiginidae**						
*Gyliotrachela hungerfordiana* (V.Moellendorff, 1886)		/	/			
*Ptychopatula orcula* (Benson, 1850)		/		/		/

# Species unique to Gunung Kuang. * Species not found in Gunung Kanthan

## Discussion

The richness of the land snail species in Gunung Kuang is much lesser than the adjacent Gunung Kanthan (47 vs 63 species). However, it is similar to other hills in Perak (47 ± 3 species, see Foon et al. 2017). Gunung Kuang is the closest hill to Gunung Kanthan, and these two hills are more than five kilometres away from other hills in this region. In view of this, we expected the two hills to share high percentage of species composition. However, only 37 out of the 63 land snail species of Gunung Kanthan were recorded in Gunung Kuang. Ten species including six unique species were found in Gunung Kuang but not in Gunung Kanthan.

**Figure 1.**
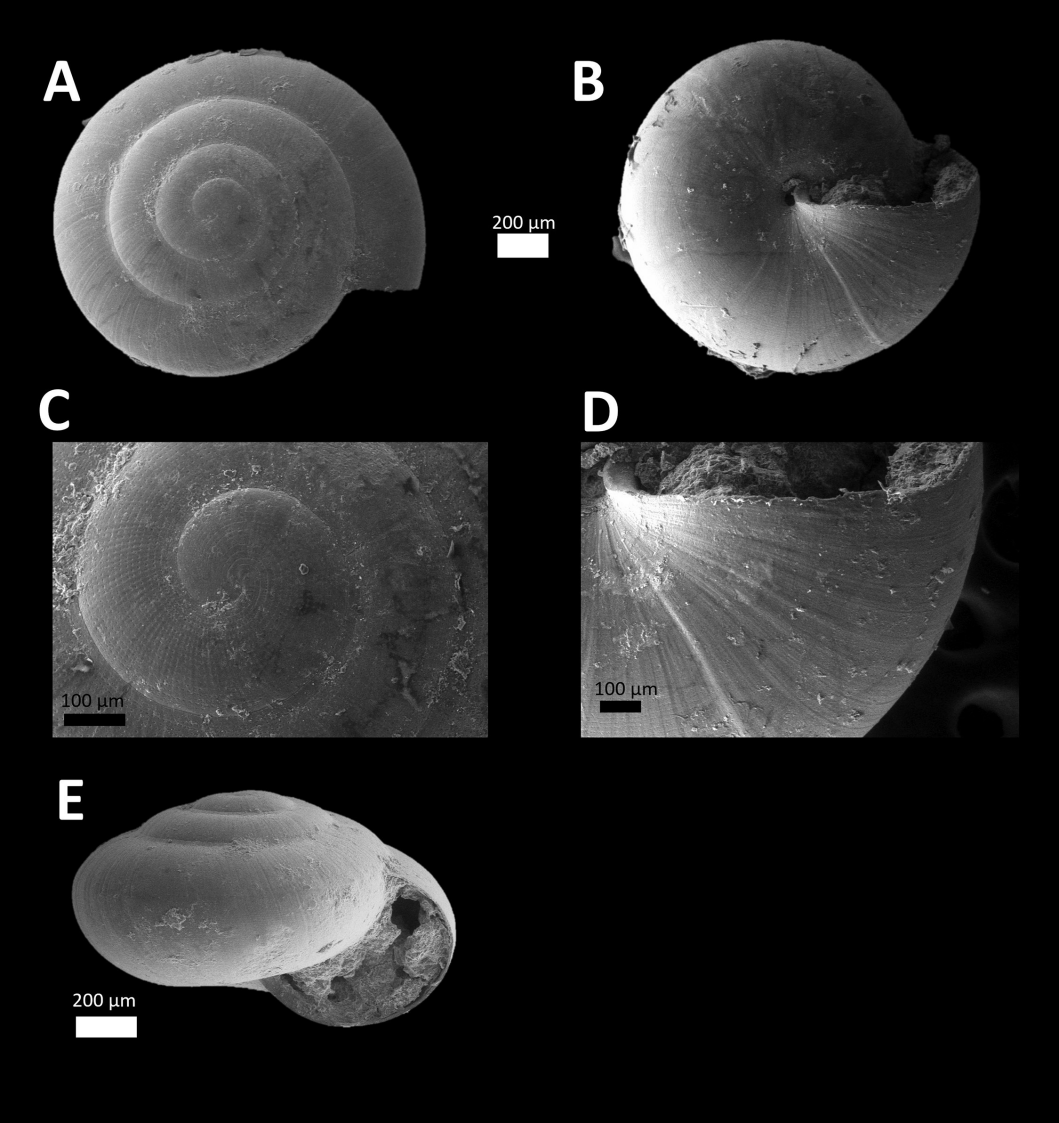
*Microcystina ‘kuang 1*’ (BORMOL 13768).

**Figure 2.**
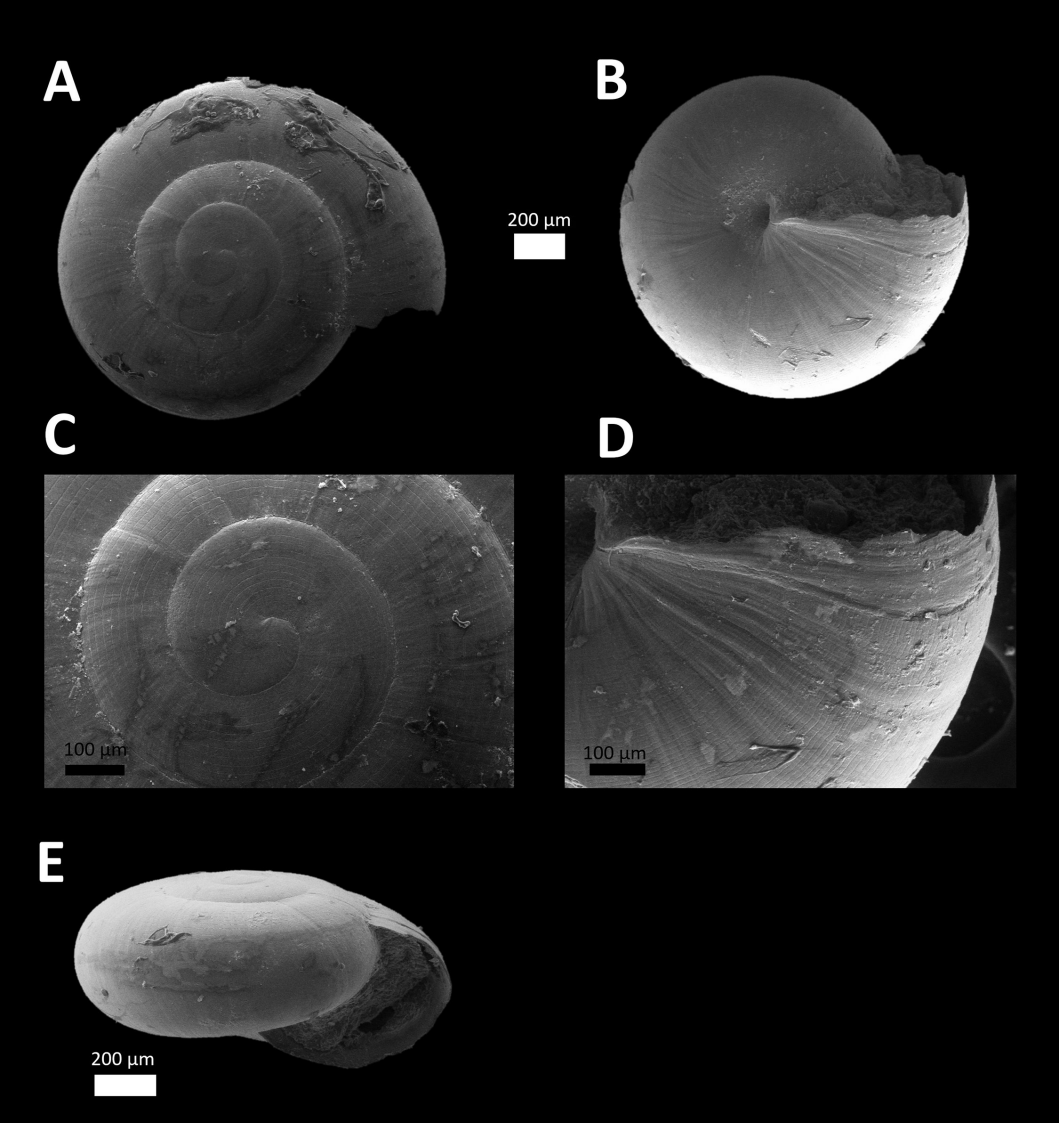
*Microcystina ‘kuang 2*’ (BORMOL 13769).

**Figure 3.**
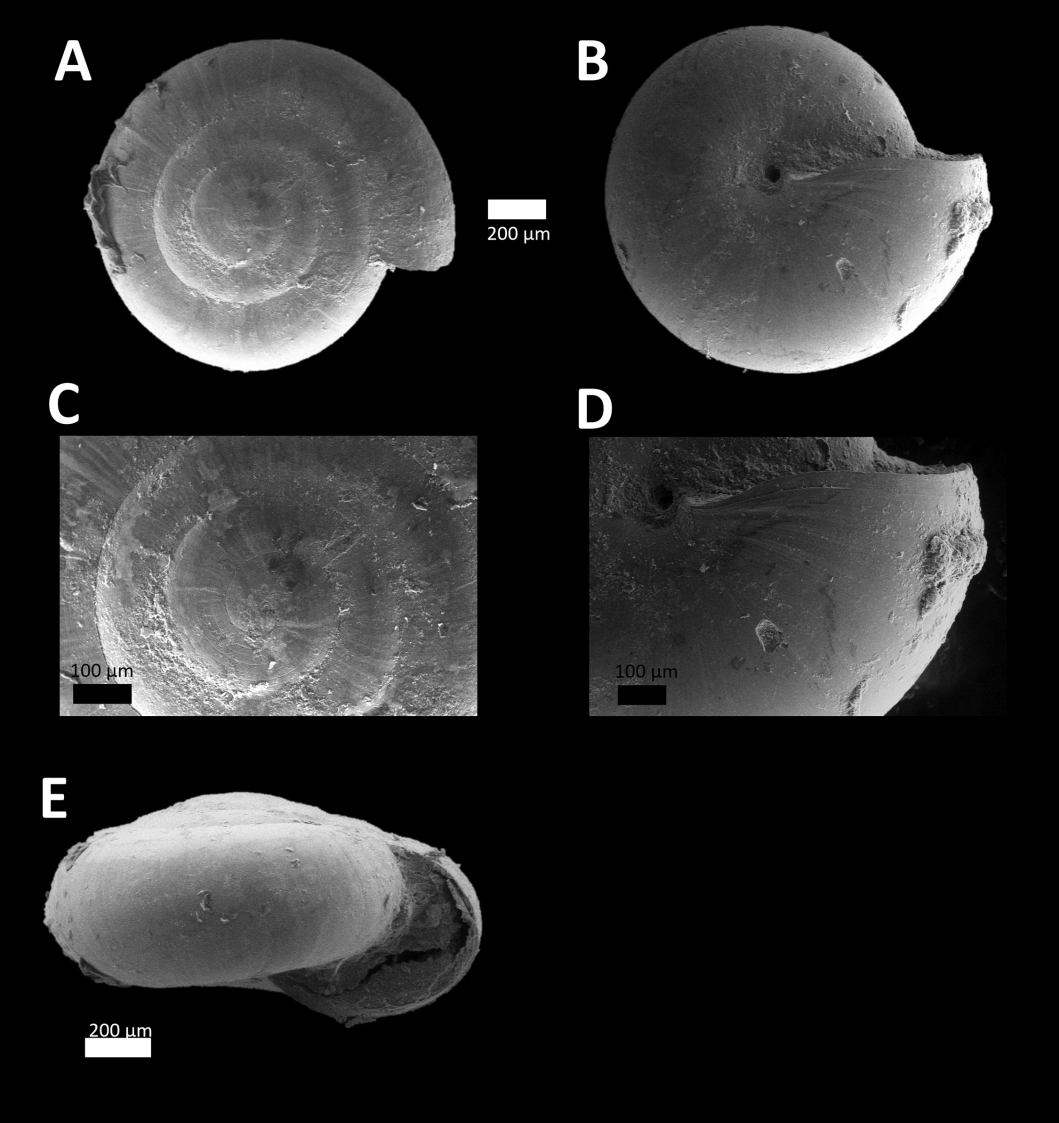
*Microcystina ‘kuang 3*’ (BORMOL 13767).

**Figure 4.**
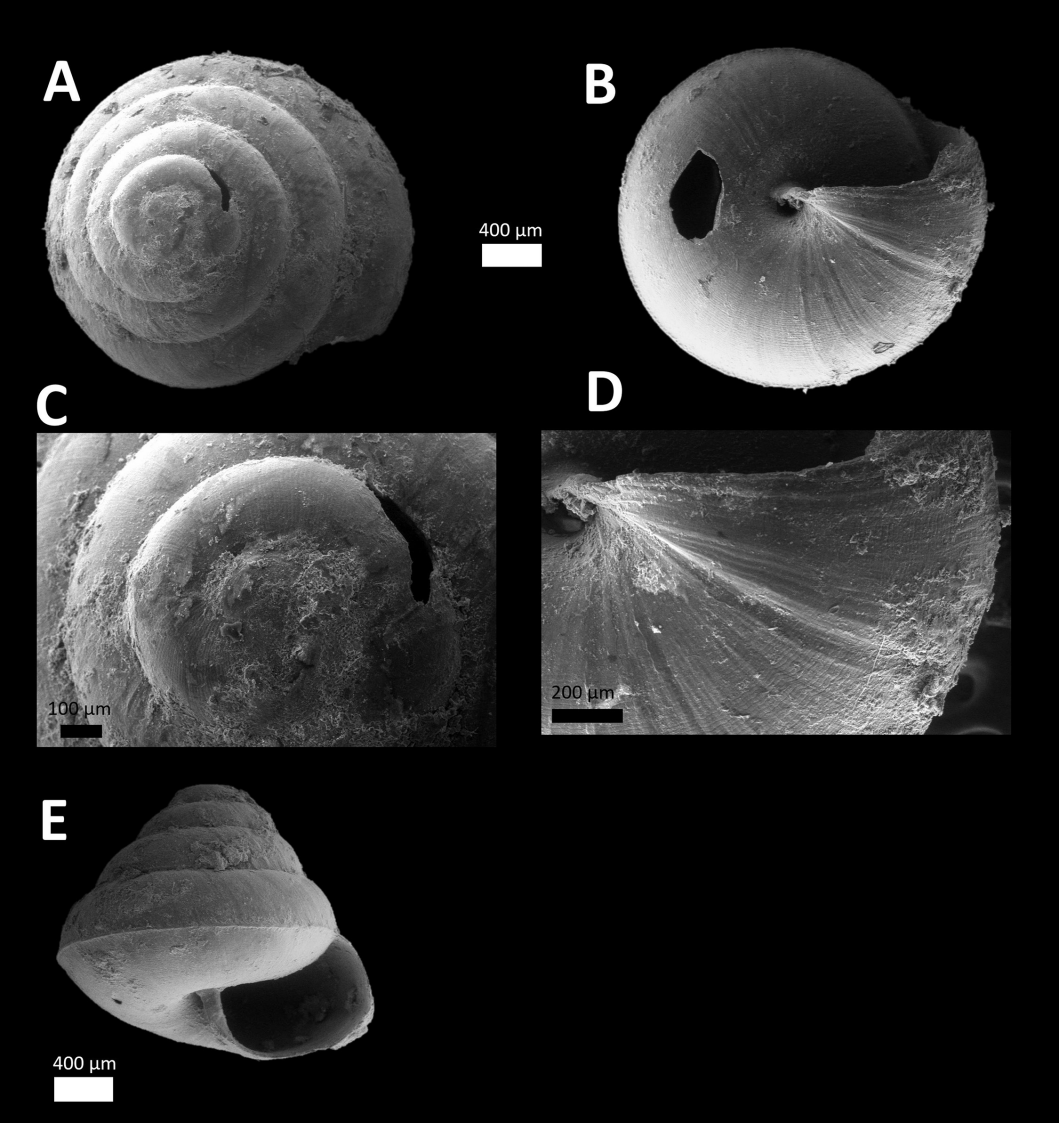
*Kaliella ‘kuang 1*’ (BORMOL 13787).

**Figure 5.**
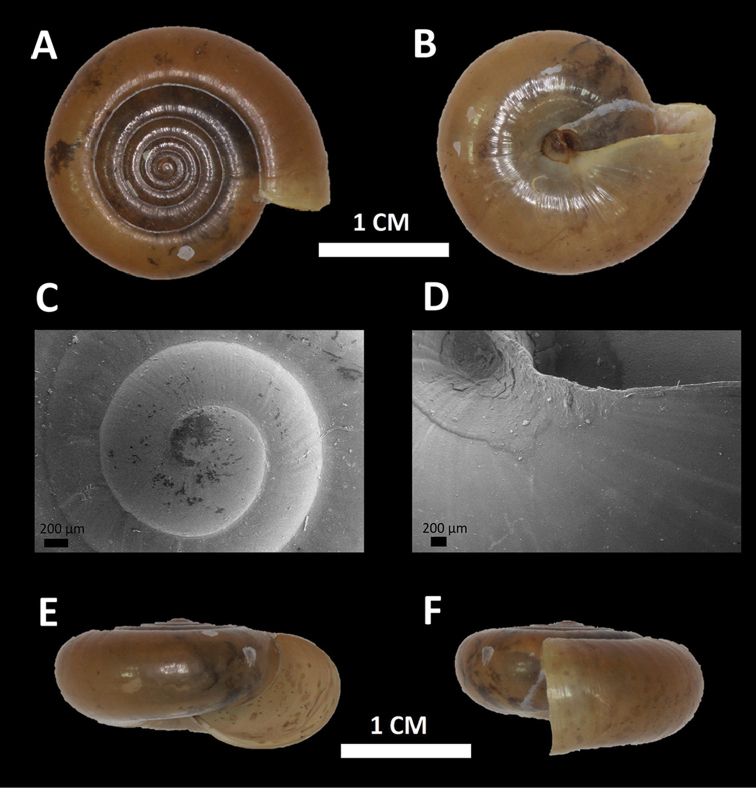
*Macrochlamys ‘kuang 1*’ (BORMOL 13784).

In addition, we found six new record of species that were neither listed in Foon et al. (2017) nor matched any known species descriptions in the literatures. The species that are unique to Gunung Kuang are *Macrochlamys ‘Kuang 1*’ (Figure 5), *Microcystina ‘Kuang 1*’ (Figure 1), *Microcystina ‘Kuang 2*’ (Figure 2), *Microcystina ‘Kuang 3*’ (Figure 3), *Kaliella ‘Kuang 1*’ (Figure 4) and *Kaliella doliolum*. Note that *Kaliella doliolum* is a widespread species, but it was not recorded from other hills surveyed in Foon et al. (2017). *Diplommatina cf. diminuta*, a previously known unique species in Gunung Kanthan, was found in Gunung Kuang. This species was also recorded from Bukit Pondok (pers. Comm. Jaap Vermeulen, collection no. V 13281). The number of unique species found in Gunung Kuang is among the highest after Prk 1 G. Tempurung and Prk 64 Bt Kepala Gajah (Foon et al. 2017).

Our survey provides additional knowledge to the land snail fauna in Kinta Valley limestone hills. Gunung Kuang is home to a high number of land snail species and also unique species. Our result shows that Gunung Kuang shares a certain degree of species composition similarity with Gunung Kanthan. This provides possible alternatives for conservation planning and the rehabilitation of certain species, particularly land snail species that are unique to the region. Nevertheless, Gunung Kanthan still requires conservation attention, as Gunung Kuang does not fully represents its species composition.

## Supplementary materials

Supplementary material 1 Species list and occurrences Link: https://doi.org/10.3897/zookeys.769.25571.suppl1 Data type: (occurrence) Explanation note: A CSV file which provided a list of species and occurrences of each species at each plot in Gunung Kuang Limestone Hill. This is an open access article distributed under the terms of the Creative Commons Attribution License (CC BY 4.0), which permits unrestricted use, distribution, and reproduction in any medium, provided the original author and source are credited.Chee-Chean Phung, Yuen-Zhao Yong, Mohamad Afandi Mat Said, Thor-Seng Liew
